# Supplementary material for: Signal-based optical map alignment
Source: PLoS One. 2021 Sep 30;16(9):e0253102. doi: 10.1371/journal.pone.0253102 (PMC8483326; doi:10.1371/journal.pone.0253102)
Supplement: S1 Fig — Obtained by fitting a quadratic function f(x) = mx2 on run-times calculated for datasets consisting of 0 to 8,000 molecules with a step of 200. The optimal value of m, obtained using non-linear least squares, was 1.34 × 10^−4^ with a standard deviation of 8.8 × 10^−7^. (PDF) [file pone.0253102.s001.pdf]

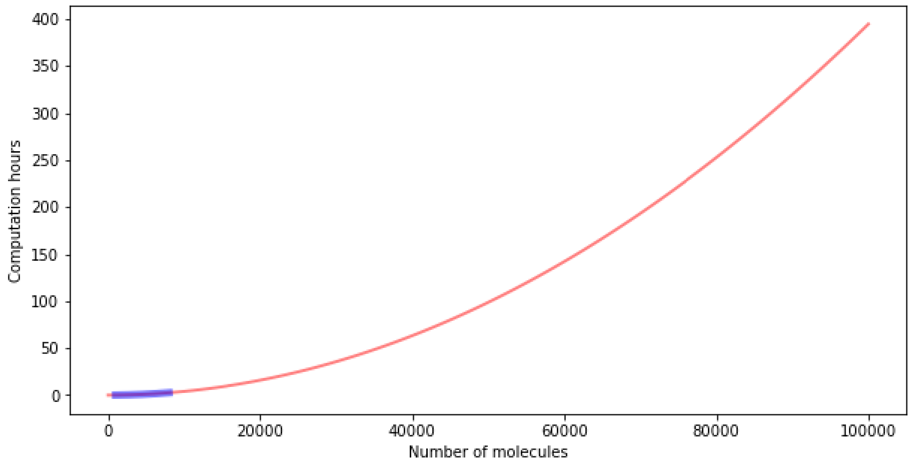

Figure S1: **Estimated time required to compute of all-vs-all OptiMap-naive alignments.** Obtained by fitting a quadratic function $f(x) = mx^2$ on run-times calculated for datasets consisting of 0 to 8,000 molecules with a step of 200. The optimal value of m, obtained using non-linear least squares, was 1.34×10^{-4} with a standard deviation of 8.8×10^{-7}.
