# Supplementary material for: Signal-based optical map alignment
Source: PLoS One. 2021 Sep 30;16(9):e0253102. doi: 10.1371/journal.pone.0253102 (PMC8483326; doi:10.1371/journal.pone.0253102)
Supplement: Attachment — Submitted filename: Response to Reviewers.pdf [file pone.0253102.s002.pdf]

Dear Editor,

We thank the reviewer for their comments and suggestions. We believe the revisions made in response to these suggestions have improved the clarity of the manuscript. Please find below a point-by-point response to the comments. The changes are marked red in the manuscript.

1. Reviewer's comment: **Fig. 5 is not used in the text.**

We thank the reviewer for picking this up. We intended to mention the figure in the text under the "Naive mode" and "Sparse overlap mode" sections. We have now added references to Fig. 5 (A, B and C) in the text (lines 199, 223, 226).

2. Reviewer's comment: **The authors used signal intensities to measure the real length between the peaks. Do the signal intensities from the peaks also reflect the number of cut sites in the pixel?**

This is a good observation. We previously studied the relationship between peak height and the number of labels in the same pixel, however we could only find a low correlation. We trained a logistic regression model on a window centered on peaks to predict whether it contained single dual labels (one or two cuts/labels within a pixel). The model's precision was 63% at a recall of 43%. Some possible explanations of the low performance can be that the peak intensities have a high level of noise and/or there are more factors involved in peak height, such as surrounding sequence or local DNA structure. Further analysis is needed for a clearer view on this relationship. We have now added some sentences suggesting that further study is needed to look into what different patterns of peaks mean (lines 320-321).

3. Reviewer's comment: **The alignment was iterated 20 times to compare OptiMap and RefAligner. Is this done on the same references with the same molecules?**

A random set of references (with corresponding molecules) as selected at each of 20 total iterations. We realise that this was not clear from the text. We have now changed the wording (lines 349-351).

4. Reviewer's comment: **In “For RefAligner, we chose 10 × 10^10 for the maximum p – value threshold”, What does this mean? How was p-value calculated for this?**

We thank the reviewer for bringing this up. The score threshold had a typo and should have $p < 10^{-10}$; and we have now fixed this in the text (line 348). For the second part of the question, we could not find any literature or software manual on how the p was calculated by the Bionano Genomics software (RefAligner).

5. Reviewer's comment: **What is (¿125 kb) in the “OptiMap performs better all-round” section?**

This was a typo and now changed to > 125kb (line 349).

6. Reviewer's comment: **In “ For OptiMap (dense mode), we used a score threshold of t = 0.65 for the naive mode (first round) and 0.55 for the sparse mode (second round)”, what does “t” mean?**

We tried to explain t as score threshold in text here:

> *"We designate a pair of molecules as an aligning pair if the score S is above the threshold (t, default 0.60) and discard the remaining pairs."*

However, we think that this was confusing and we replaced t altogether and used the following notation: "$S > 0.65$" throughout the paper to denote score threshold (lines 208, 229, 237, 243, 346, 347, 357).

7. Reviewer's comment: **Are the graphs in Fig 10 made with Optiscan or BGN (Are the data stretched)?**

We thank the reviewer for their attention to the figure. The figures were made from the perspective of Optimap, after linear stretching, as it shows where Optimap goes wrong (Fig 10 B.: in the top example there is local stretching just after 200bp and in the bottom example after 150bp). This is now explained in the figure caption.
